# Book Reviews

**Published:** 1893-10

**Authors:** 


### Book Reviews.

A NEW ILLUSTRATED DICTIONARY OF MEDICINE, BIOLOGY, AND COLLATERAL SCIENCES.—Dr. George M. Gould, already well-known as the editor of two small medical dictionaries, has now about ready an unabridged, exhaustive work of the same class, upon which he and a corps of able assistants have been uninterruptedly engaged for several years.

The feature that will attract immediate attention is the large number of fine illustrations that have been included, many of which as, for instance, the series of over fifty of the bacteria—have been drawn and engraved especially for the work. Every scientific-minded physician will also be glad to have defined several thousand commonly used terms in biology, chemistry, etc.

The chief point, however, upon which the editor relies for the success of his book is the unique epitomization of old and new knowledge. It contains a far larger number of words than any other one-volume medical lexicon. It is a new book, not a revision of the older volume. The pronunciation, etymology, definition, illustration, and logical groupings of each word are given. There has never been such a gathering of new words from the living literature of the day. It is especially rich in tabular matter, a method of presentation that focuses, as it were, a whole subject so as to be understood at a glance.

The latest method of spelling certain terms, as adopted by various scientific bodies and authorities, have all been included, as well as those words classed as obsolete by some editors, but still used largely in the literature of to-day, and the omission of which in any work aiming to be complete would make it unreliable as an exhaustive work of reference.

The publishers announce that, notwithstanding the large outlay necessary to its production on such an elaborate plan, the price will be no higher than that of the usual medical text-book.

THE BACTERIAL POISONS, by DR. N. GAMALEIA. Translated by E. P. Hurd, M. D., Member of the Massachusetts Medical Society, and of the Climatological Society; one of the Physicians to the Newburyport (Mass.) Hospital. George S. Davis, 1893.

This number of the Physicians' Leisure Library is of interest at the present time when our seaports are threatened with both cholera and yellow fever, and the qustion of *bacteria* will be discussed.

---

INTERNATIONAL CLINICS, A QUARTERLY OF CLINICAL LECTURES ON MEDICINE, NEUROLOGY, PEDIATRICS, SURGERY, GENITO-URINARY SURGERY, GYNÆCOLOGY, OPHTHALMOLOGY, LARYNGOLOGY, OTOLOGY AND DERMATOLOGY, by Professors and Lecturers in the Leading Medical Colleges of the United States, Great Britain and Canada. Vol. I. Third Series, 1893. Philadelphia, J. B. Lippincott Co.

The INTERNATIONAL CLINICS are now too generally read by the profession to need any commendation. This the first volume of the third series is up to the high standard of the preceding volumes, and contains many articles of lasting interest to the profession.

---

About October 15th a Medical Directory of the State of Connecticut will be issued by the Danbury Medical Printing Co., of Danbury, Conn. It will contain a list of all the medical practitioners of the State, the various medical societies, all the dentists and dental societies, druggist and pharmaceutical societies, nurses and training schools for nurses, hospitals, etc. Price $1, delivered free by post.

---

Dr. Thomas More Madden, well known as one of the foremost gynæcologists of Europe, has prepared a hand-book of Diseases Peculiar to Women, which will be published at once by J. B. Lippincott Company. The work is more than usually important from the fact that it contains the result of the author's wide experience and is embellished with numerous excellent sketches of gynæcological diseases and appliances, together with engravings from drawings or photographs of cases under clinical observation made by some of the most eminent physicians in England and America. The work is entitled, "Clinical Gynæcology."

